# The complete mitogenome of *Microhyla fissipes* (Anura: Microhylidae) and phylogenetic analysis using GenBank data mining

**DOI:** 10.1080/23802359.2019.1666670

**Published:** 2019-09-18

**Authors:** Ning Han, Zhaoqing Wu, Luxin Zhang, Xuhui Wei

**Affiliations:** Jincheng College of Sichuan University, Chengdu, China

**Keywords:** *Microhyla fissipes*, complete mitochondrial genome, phylogeny

## Abstract

The complete mitogenome of *Microhyla fissipes* (16,723 bp) was obtained and analysed. It contains the set of 13 protein-coding genes, two rRNA genes, 22 tRNA genes, and one non-coding regions. Most of the genes in *M. fissipes* are located on the H-strand, except for the ND6 and eight tRNA genes which are located on the L-strand. The phylogenetic tree shows that *M. fissipes* is a sister to the clade composed of *M. okinavensis* and *M. mixtura* and places *Glyphoglossus yunnanensis* as the sister lineage to a clade of *Microhyla*. This new mitogenome of *M. fissipes* will provide basic data for further molecular evolution studies in this genus.

The ornamented pygmy frog *Microhyla fissipes* (Anura: Microhylidae) is broadly distributed in southern and central China (including Taiwan and Hainan Island) northeast of the Red River Valley (from southern Yunnan northward and east to Shanxi and Shaanxi) (Frost 2019). To date, several species (*M. butleri*, *M. heymonsi*, *M. mixture*, *M. okinavensis*, *M. pulchra,* and *M. taraiensis*) of this genus have been determined the complete mitogenome sequence and the related phylogenetic relationships have also been investigated (Zhang et al. 2005; Wang et al. 2016; Wu et al. 2016; Yong et al. 2016; Khatiwada et al. 2018; Zhao et al. 2018). Herein, we use mitogenome sequence under Maximum-Likelihood (ML) and Bayesian inference (BI) criteria to elucidate the relationship of *M. fissipes* to other *Microhyla*.

The specimen of *M. fissipes* was collected from Yaan in Sichuan, (29°58′43.46″N and 102°59′4.57″E; elevation 660 m asl) in May 2018 and stored in Museum of Jincheng College of Sichuan University (Specimen voucher No. JC2018001). Small pieces of muscle tissue were taken and preserved in absolute ethanol. Total genomic DNA was extracted using Ezup-pillar Genomic DNA Extraction Kit (Sangon, Shanghai, China). DNA sample was sent to Personal Biotechnology Co, Ltd (Shanghai, China) for library construction and sequencing using the IIlumina Miseq System (Metzker 2010). We analyzed the complete mitogenome of *M. fissipes* and performed phylogenetic analyses for the new obtained and the other related Microhylidae frogs mitogenomes that available in GenBank.

The mitogenome of *M. fissipes* is 16,723 bp long (GenBank accession no. MN046210), comprising 13 protein-coding genes, two ribosomal RNAs, and 22 transfer RNAs genes, along with a large control region (D-loop). The total A + T content of this mitogenome is 59.94%, with base compositions of 28.93%A, 31.01%T, C 25.48%C, and G 14.58%G. ND6 gene and eight tRNA genes (trnQ, trnA, trnN, trnC, trnY, trnS2, trnE, and trnP) were encoded in the L-strand, whereas the rest of genes were encoded in the H-strand. The tRNAs ranged from 66 to 74 bp in size, and the length of 12S rRNA and 16S rRNA are 938 and 1,577 bp, respectively. In addition, the control region (1,307 bp) is flanked by cyt *b* and tRNA-L1 genes. The gene arrangement pattern and transcription directions were concordant with those previous studies in *M. taraiensis* (Khatiwada et al. 2018).

Sixteen mitogenome sequences including 14 Microhylidae and *Babina adenopleura* and *Pelophylax chosinica* were used for phylogenetic analyses, with setting the latter two species as the outgroups. Phylogenetic trees were reconstructed under both MEGA X (Kumar et al. 2018) and MrBayes v3.2 (Ronquist et al. 2012) using maximum-likelihood (ML) and Bayesian inference (BI) methods, respectively. ML and BI phylogenetic trees (Figure 1) based on the concatenated nucleotide sequences of 13 protein-coding genes showed identical topology and reveal that *M. fissipes* is a sister to the clade composed of *M. okinavensis* and *M. mixtura* and places *Glyphoglossus yunnanensis* as the sister lineage to a clade of *Microhyla* with a bootstrap value of 98 (Figure 1). The results of phylogenetic relationships in Microhylidae here are congruent with previous study (Khatiwada et al. 2018).

**Figure 1.**
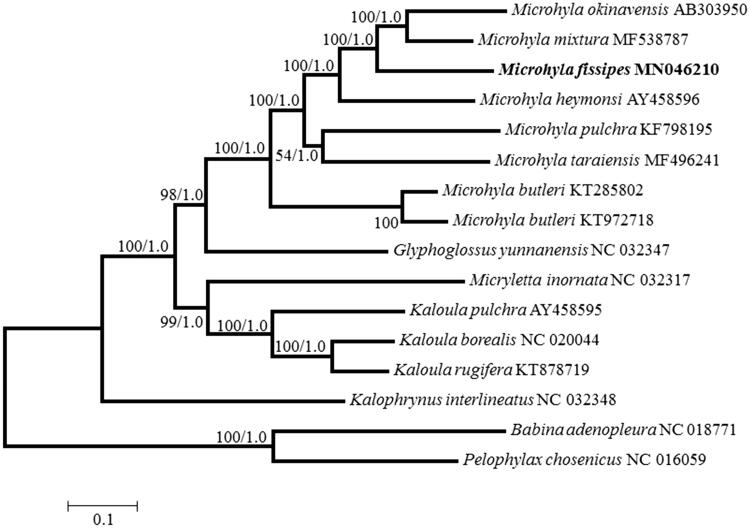
Maximum-likelihood (ML) and Bayesian inference (BI) phylogenetic tree of sixteen frog species based on 13 protein-coding genes. ML bootstraps and BI posterior probabilities are shown at the node. The GenBank accession numbers are listed following species names.

## References

[CIT0001] Frost D 2019 Amphibian species of the world: an Online Reference. Version 6.0. American Museum of Natural History, New York, USA: American Museum of Natural History; [accessed 2019 June 13]. http://research.amnh.org/herpetology/amphibia/index.html.

[CIT0002] Khatiwada JR, Wang S, Shu G, Xie F, Jiang J 2018 The mitochondrial genome of the *Microhyla taraiensis* (Anura: Microhylidae) and related phylogenetic analyses. Conservation Genet Resour. 10:441–444.

[CIT0003] Kumar S, Stecher G, Li M, Knyaz C, Tamura K 2018 MEGA X: molecular evolutionary genetics analysis across computing platforms. Mol Biol Evol. 35:1547–1549. 29722887 10.1093/molbev/msy096 PMC5967553

[CIT0004] Metzker ML 2010 Sequencing technologies – the next generation. Nat Rev Genet. 11:31–46. 19997069 10.1038/nrg2626

[CIT0005] Ronquist F, Teslenko M, Van Der Mark P, Ayres DL, Darling A, Höhna S, Larget B, Liu L, Suchard MA, Huelsenbeck JP 2012 MrBayes 3.2: efficient Bayesian phylogenetic inference and model choice across a large model space. Syst Biol. 61:539–542. 22357727 10.1093/sysbio/sys029 PMC3329765

[CIT0006] Wang S, Liu L, Jiang J 2016 The complete mitochondrial genome of *Microhyla butleri* (Amphibia, Anura, Microhylidae). Mitochondrial DNA B. 1:154–155. 10.1080/23802359.2016.1144107 PMC7800870 33473443

[CIT0007] Wu X, Li Y, Zhang H, Yan L, Wu X 2016 The complete mitochondrial genome of *Microhyla pulchra* (Amphidia, Anura, Microhylidae). Mitochondrial DNA A DNA Mapp Seq Anal. 27:40–41. 24438273 10.3109/19401736.2013.869685

[CIT0008] Yong H, Song S, Lim P, Eamsobhana P, Tan J 2016 Complete mitochondrial genome and phylogeny of *Microhyla butleri* (Amphibia: Anura: Microhylidae). Biochm Syst Ecol. 66:243–253.

[CIT0009] Zhang P, Zhou H, Chen Y, Liu Y, Qu L 2005 Mitogenomic perspectives on the origin and phylogeny of living amphibians. Syst Biol. 54:391–400. 16012106 10.1080/10635150590945278

[CIT0010] Zhao Y, Meng H, Su L 2018 The complete mitochondrial genome of the mixtured pygmy frog *Microhyla mixtura* (Anura, Microhylidae). Conservation Genet Resour. 10:427–430.

